# The Impact of Hypoxia on the Host-Pathogen Interaction between Neutrophils and *Staphylococcus aureus*

**DOI:** 10.3390/ijms20225561

**Published:** 2019-11-07

**Authors:** Natalia H Hajdamowicz, Rebecca C Hull, Simon J Foster, Alison M Condliffe

**Affiliations:** 1 Department of Infection, Immunity and Cardiovascular Diseases, University of Sheffield, Beech Hill Road, Sheffield S10 2TN, UK; nhhajdamowicz1@sheffield.ac.uk (N.H.H.); rchull1@sheffield.ac.uk (R.C.H.); 2 Florey Institute, University of Sheffield, Beech Hill Road, Sheffield S10 2RX, UK; s.foster@sheffield.ac.uk

**Keywords:** neutrophils, host-pathogen interaction, hypoxia, *Staphylococcus aureus*

## Abstract

Neutrophils are key to host defence, and impaired neutrophil function predisposes to infection with an array of pathogens, with *Staphylococcus aureus* a common and sometimes life-threatening problem in this setting. Both infiltrating immune cells and replicating bacteria consume oxygen, contributing to the profound tissue hypoxia that characterises sites of infection. Hypoxia in turn has a dramatic effect on both neutrophil bactericidal function and the properties of *S. aureus*, including the production of virulence factors. Hypoxia thereby shapes the host–pathogen interaction and the progression of infection, for example promoting intracellular bacterial persistence, enabling local tissue destruction with the formation of an encaging abscess capsule, and facilitating the establishment and propagation of bacterial biofilms which block the access of host immune cells. Elucidating the molecular mechanisms underlying host–pathogen interactions in the setting of hypoxia will enable better understanding of persistent and recalcitrant infections due to *S. aureus* and may uncover novel therapeutic targets and strategies.

## 1. Introduction

Our understanding of host–pathogen interactions has gradually evolved, from the initial concept of an invader overcoming or being defeated by host defences to a more complex and flexible relationship with microbes, where outcomes may result in damage, benefit or be entirely neutral. There are thus a range of ‘interaction’ states including colonisation, commensalism and disease, and the same microbe may shift between these states depending on the precise microenvironment in which the interaction between host and microbe occurs. In this review, we explore the impact of reduced oxygen availability (‘hypoxia’) on the interaction between *Staphylococcus aureus* (*S. aureus*) and our most abundant innate immune cell, the neutrophil.

*S. aureus* is a Gram-positive coccal bacterium, which colonises around 30% of the population worldwide [1]. It causes frequent localised infections of the skin, but can also induce life-threatening human infection if mucosal surfaces are breached. *S. aureus* possesses numerous virulence factors which help it cross the skin barrier and access deeper tissues, thereby causing a spectrum of diseases from local skin and soft tissue infections (SSTI including superficial abscesses, wound infections, and cellulitis) to more severe and invasive conditions with a high morbidity and mortality, such as osteomyelitis, joint infections, endocarditis and bacteraemia/septicaemia [2]. Invasive infection may be enhanced by the ability of other skin commensals to act as ‘pro-infectious agents’, dramatically reducing the *S. aureus* infectious dose required to initiate disease [3]. *S. aureus* has a propensity to infect medical devices, including intravenous cannulae [4] and prosthetic joints or heart valves [5], promoted by its ability to form biofilms. The importance of *S. aureus* as a human pathogen is enhanced by its multidrug resistance profile; there are strains resistant to most available antibiotics e.g., methicillin-resistant *S. aureus* (MRSA) and vancomycin-resistant *S. aureus* (VRSA) [6].

In addition to its ability to respond to evolutionary pressures from antibiotics, *S. aureus* has also evolved to combat professional phagocytic cells such as polymorphonuclear leukocytes (PMNs or neutrophils). Neutrophils are crucial for human defence against staphylococcal infections, as highlighted when neutrophil function is defective (see below). However, *S. aureus* may lyse the engulfing phagocyte, or persist inside even fully competent neutrophils despite the high-grade bactericidal functions these cells command [7]. Many factors contribute to the resistance of *S. aureus* to host-mediated killing, including the propensity of this pathogen to infect areas of relative tissue hypoxia. Whilst in healthy tissues, the oxygen tension is commonly 20–70 mm Hg (2.5–9% O_2_), infection sites show much lower oxygen levels <10 mm Hg (<1% oxygen) [8]. The efficacy of innate immune cells to handle this pathogen is thus at least partly dependent on their ability to operate in a low-oxygen milieu and overcome *S. aureus* adaptations to the local immune and environmental pressures.

There is incomplete understanding of interactions between innate immune cells and *S. aureus*, and how these interactions may result in pathogen death, containment or dissemination. Studying host–pathogen interaction under conditions that resemble those present in humans during bacterial invasion, such as hypoxia, will promote better understanding of how this important pathogen establishes and perpetuates infection.

## 2. Neutrophil Killing Mechanisms

**Figure 1:**
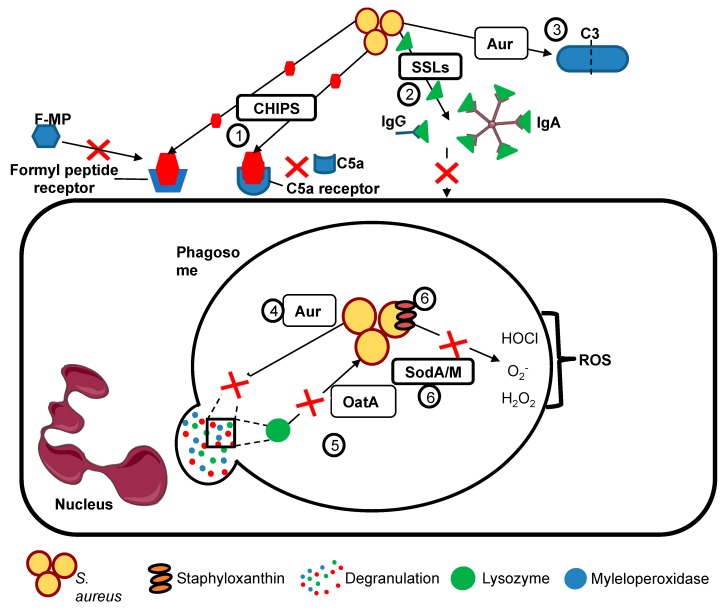
*S. aureus* avoids engulfment and killing by neutrophils. *S. aureus* avoids killing by neutrophils by preventing phagocytosis and resisting internal killing mechanisms using a number of strategies, including: (**1**) Neutrophil chemotaxis is inhibited by chemotaxis inhibitory protein of *S. aureus* (CHIPS), which prevents binding of chemoattractants such as activated complement and bacterial formylated peptides (F-MP) to neutrophil C5a and formyl peptide receptors. (**2**) Staphylococcal superantigen-like proteins (SSLs) bind IgG and IgA preventing their adherence to neutrophils and hence blocking opsonisation. Aureolysin prevents (**3**) complement activation by cleaving C3, blocking C3a activation. Granule-derived antimicrobial peptides such as lysozyme or MPO myeloperoxidase are also cleaved by (**4**) aureolysin (Aur). (**5**) *S. aureus* is protected from degradation by lysozymes through modification of peptidoglycan by O-acetyltransferase (OatA). (**6**) There are multiple systems to combat ROS including antioxidants such as SodA/SodM and Staphyloxanthin, which protect staphylococcus from the oxidative stress due to ROS. This figure was created using Servier Medical Art templates, which are licensed under a Creative Commons Attribution 3.0 Unported License; https://smart.servier.com.

Neutrophils are rapidly responsive, but short-lived, bone marrow-derived innate immune cells. They are the most abundant circulating leucocytes, crucial to the host response against invading pathogens. Neutrophils are not tissue residents but transmigrate on demand to the site of an infection, where they phagocytose and kill invading pathogens, by deployment of pre-formed antimicrobial granules, by the de novo generation of reactive oxygen species (ROS) and by the release of chromatin decorated with histones and granule proteins such as myeloperoxidase (NETosis); these mechanisms and how they are modulated by hypoxia are discussed in more detail below. *S. aureus* combats these innate immune functions by the release of a range of virulence factors, examples of which are shown in Figure 1 and Table 1. These include (but are not limited to) staphylococcal superantigen-like (SSL) proteins, which bind immunoglobulins and complement components to block opsonisation, and chemotaxis inhibitory protein of *S. aureus* (CHIPS), which prevents binding of chemoattractants such as formylated peptides to neutrophil receptors (Figure 1).

Micro-organisms present a range of pathogen-associated molecular patterns (PAMPs) and are recognised by receptors, e.g., toll-like receptors (TLRs), which are present on the surface of immune cells including neutrophils [9]. PMNs recognise multiple pathogen-associated molecules whether integral or secreted by Gram-positive or Gram-negative bacteria. These include peptidoglycan (sensed by multiple pattern-recognition receptors, including nucleotide-binding oligomerization domain-containing protein 1 (NOD1), NOD2, NOD-, LRR- and pyrin domain-containing 3 (NLRP3) and peptidoglycan recognition protein 1 (PGLYRP1)), lipoteichoic acids (Toll-like receptor (TLR) 2 ligand), lipopolysaccharide (recognised by the TLR4 and myeloid differentiation factor 2 (MD-2) complex) or flagellin (TLR5 ligand). Other bacterial products including formylated peptides (F-MPs: the exemplar is *N*-formyl-methionyl-leucyl-phenylalanine, fMLP) interact via dedicated formyl peptide receptors to recruit neutrophils to the site of infection and subsequently activate their bactericidal functions. Opsonins such as complement fragments and antibodies bind the pathogen and interact with phagocyte receptors. Pathogen recognition is essential for subsequent phagocytosis, which is further facilitated by opsonisation.

Phagocytosis is a complex process linking cell surface receptors to alterations in membrane conformation and the actin cytoskeleton. Phagocytosed pathogens are enclosed in a membrane-bound phagosome, within which most (but not all) microorganisms are effectively killed (Figure 1) by the combination of degranulation of antimicrobial agents (from pre-formed neutrophil granule populations including a range of proteases and enzymes such as elastase, myeloperoxidase (MPO) and the cathepsins, iron sequestrators such as lactoferrin, and a plethora of other bactericidal molecules) and by the assembly of the NADPH oxidase complex at the phagosomal membrane enabling ROS production, the ‘oxidative burst’, and formation of reactive nitrogen species such as nitric oxide (NO). If the internalisation of pathogen is not feasible, neutrophils may release NETs in an an attempt to kill invaders extracellularly; however this strategy is not always successful [10].

## 3. Neutrophil Dysfunction and Staphylococcal Infection

Several inherited and acquired neutrophil disorders have been described (examples listed in Table 2; [11,12,13,14,15,16,17,18,19]), often leading to life-threating infections; *S. aureus* is a prominent pathogen in these settings, suggesting neutrophils are key in restricting its pathogenicity. Patients with such defects often receive continuous anti-staphylococcal antibiotic prophylaxis.

The importance of the neutrophil in the anti-staphylococcal immunity was highlighted when chronic granulomatous disease (CGD) was first described and its cause identified [20]. Neutrophils from CGD patients are unable to produce ROS from molecular oxygen, because of mutations in components of NADPH oxidase enzyme. CGD patients are highly susceptible to infections caused by microorganisms such as *Staphylococcus* spp., *Aspergillus* spp., *Salmonella* spp., and *Serratia* spp. [21], as the defective oxidative burst leads to a failure of pathogen killing. Other disorders of neutrophil function likewise confer susceptibility to *S. aureus* infection [11,12,13,14,15,16,17,18,19], underscoring the importance of these cells in host–pathogen interactions.

## 4. Physiological and Pathological Hypoxia

Hypoxia represents an imbalance between oxygen supply and demand. Oxygen gradients exist within and across tissues; this ‘physiological hypoxia’ is heightened by disease processes such as inflammation and infection, leading to ‘pathological hypoxia’. Local tissue ‘hypoxia’ is in fact normal in the healthy organism. The oxygen level in tissue environments differs considerably from that of inspired air (*p*O_2_ about 160 mmHg). Circulating neutrophils repeatedly transit between a *p*O_2_ of approximately 100 mmHg in main arteries, 50 mmHg in arterioles and 20–30 mmHg in capillaries. Since oxygen must diffuse from capillaries to the surrounding environments, the oxygen tension in normal tissues can be even lower, leading to ‘physiological hypoxia’. Tissue oxygenation can be measured using micro-electrodes or by staining with compounds such as pimonidazole, which binds to thiol groups at oxygen tensions below 10 mmHg, and the OxyLite PO_2_ system (which determines the O_2_-dependent fluorescent lifetime of ruthenium chloride); for example, the *p*O_2_ in the healthy thymus is approximately 10 mm Hg and around 16 mm Hg in the spleen [22].

Tissue oxygenation status may be compromised by a range of pathological processes, such as atherosclerosis (reduced delivery of oxygenated blood by narrowed vessels) and malignancy (abnormal blood vessels and increased diffusion distance). Importantly, neutrophilic infiltration induces ‘inflammatory hypoxia’. In a sterile colitis model, infiltrating PMNs depleted local epithelial oxygen levels by consuming oxygen to fuel the oxidative burst [23]. In the setting of infection, bacteria also contributes to tissue hypoxia, for example *S. aureus* depleted dissolved oxygen in a skin infection model [24] and stabilisation of HIF1α (a marker of hypoxia, see below) has been demonstrated in human skin biopsies in both keratinocytes and infiltrating neutrophils in the setting of *S. aureus* skin infection [25]. Together, neutrophilic inflammation and bacterial replication contribute to the establishment of ‘pathological hypoxia’.

Thus, neutrophils must sense local oxygen levels and adapt to operate within hypoxic environments in order to control infection by *S. aureus*; hence it is important to establish the impact of hypoxia on key neutrophil microbicidal functions and on bacterial virulence determinants. Of note, most data on neutrophil and bacterial function have been collected by studying isolated cells cultured in atmospheric oxygen (160 mmHg), far in excess of the oxygen levels in the relevant tissue environment.

## 5. Mammalian Oxygen Sensing and Response to Hypoxia

The mammalian molecular response to hypoxia is largely mediated by the family of hypoxia-inducible factor (HIF) transcription factors and has been summarised in detail elsewhere [26]. In brief, HIF-α stability is post-transcriptionally regulated by molecular oxygen, via oxygen-sensitive prolyl hydroxylases (PHDs). There are three HIFs and three PHDs, and it is now appreciated that they have somewhat differing roles in regulating neutrophil function and metabolic responses in hypoxic settings [27]. When oxygen is available, PHDs are catalytically active and hydroxylate HIF-α, targeting it for proteasomal degradation. Another oxygen-dependent enzyme, Factor Inhibiting HIF (FIH), hydroxylates asparagine residues to block association with transcriptional co-activators CBP/p300 [28]. When molecular oxygen in the cytoplasm is reduced, PHD and FIH are inactive, HIF-1α subunits accumulate in the cytoplasm, heterodimerise with constitutively expressed HIF-1β and recruit p300/CBP coactivator proteins. This regulatory complex transmigrates to the nucleus and binds to hypoxia responsive elements (HREs) to control the transcription of target genes which enact cellular hypoxic responses.

Although HIFs are often referred to as the master regulators of hypoxic responses, it is important to note that HIFs can also be activated by inflammatory signals independent of hypoxia (for example, bacterial lipopolysaccharide mediated a transcription-dependent upregulation of HIF-1α expression in macrophages [29]), and it is increasingly recognised that some effects of hypoxia are independent of HIFs (e.g., hypoxia-enhanced degranulation [30]). Hence a neutrophil migrating into inflamed tissues will experience variable oxygen tensions, leading to a range of HIF-dependent and HIF-independent effects in a dynamic and context-dependent fashion.

## 6. *S. aureus* Responses to Adverse Environmental Conditions

**Figure 2:**
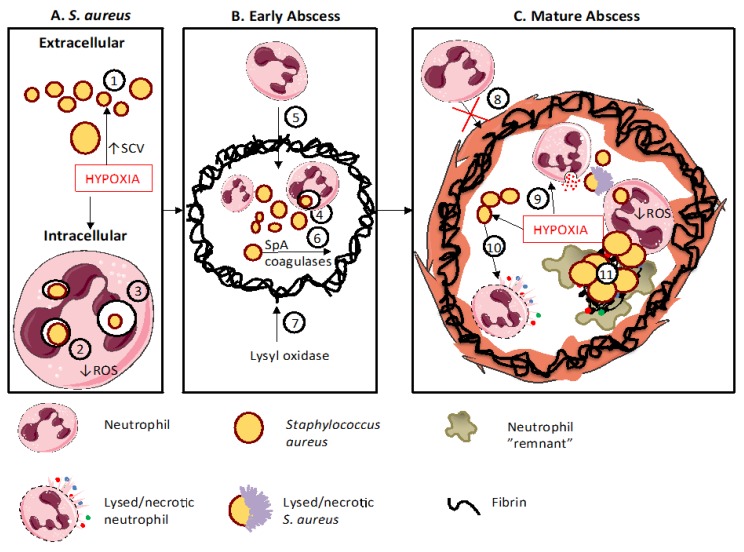
Neutrophils and hypoxia contribute to abscess formation and development. Panel A depicts the interaction between neutrophil and *S. aureus* in a typical hypoxic environment, panel B the early stages in abscess formation and panel C the role of hypoxia in establishing the mature abscess and preventing the resolution of infection and inflammation. **A.** A hypoxic micro-environment promotes the emergence of small colony variants (SCVs, 1) which, together with decreased neutrophil ROS generation consequent to a lack of molecular oxygen (2) promotes enhanced intracellular persistence (3). **B.** An early abscess develops from an extracellular bacterium or from an intracellular *S. aureus* (4), which has been carried from a distal site. Neutrophils can enter the early abscess, (5) but formation of a fibrin capusle is instigated by *S. aureus* factors such as SpA and coagulases (6) and by HIF-dependent lysyl oxidase from surrounding cells (7). **C.** Once formed, the mature abscess capsule prevents further neutrophil infiltration (8). The highly hypoxic conditions alter neutrophil processes by enhancing degranulation (9) and enhanced *S. aureus* secretion of leukocidins (10) to induce neutrophil lysis. The mature abscess contains products from necrotic and degranulated neutrophils, multiplying *S. aureus* and persisting intracellular *S. aureus* within neutrophils (11). This figure was created using Servier Medical Art templates, which are licensed under a Creative Commons Attribution 3.0 Unported License; https://smart.servier.com.

Pathogens such as *S. aureus* evolve to exploit local conditions and circumvent immune-mediated killing, with the emergence of bacterial strains possessing a wide range of virulence factors; it is thus of importance to study the host–pathogen interaction including such virulent bacterial strains rather than concentrating on single laboratory-adapted strains. Staphylococcal virulence factors (see Table 1) have been reviewed in detail elsewhere [31] and include substances that combat phagocyte mobilisation, recognition and phagocytosis; toxins to destroy host immune cells, factors which detoxify host bactericidal peptides, proteases and ROS; immune modulators that enable phagosomal escape; and elements to allow adaptation to adverse conditions such as hypoxia (see below). Several *S. aureus* strains can survive intracellularly (Figure 2), either by reducing virulence (Small Colony Variants or SCVs, discussed in more detail below), by ‘defusing’ the phagosomal arsenal (eg by secretion of catalase) or by escaping from the phagosome (reviewed in [32]); such adaptations may be driven by external conditions, either in the extracellular or intracellular environment. *S. aureus* has been found to remain viable and virulent inside murine PMNs, within larger vacuoles called “spacious phagosomes” or inside small vacuoles termed “tight phagosomes” [33]; those within spacious phagosomes (suggested to have formed via macropinocytosis rather than true phagocytosis) seemed more likely to survive, perhaps due to failure to deploy the normal killing mechanisms in this setting. Hypoxic neutrophils or neutrophils isolated from patients with CGD were able to contain, but not kill ingested *S. aureus* [34], again highlighting the importance of the oxidative burst in the execution of this pathogen.

In common with many bacteria, *S. aureus* senses and responds to environmental changes via two-component regulatory systems, comprising a histidine kinase that senses a specific environmental stimulus and a corresponding response regulator, which controls the differential expression of target genes. *S. aureus* controls the production of virulence factors through two-component regulatory loci including *sae* (*S. aureus* exoprotein expression), *agr* (accessory gene regulator), and also *ssrAB* (staphylococcal respiratory response AB) and air*SR* (anaerobic iron-sulfur cluster-containing redox sensor regulator); the latter two systems sense hypoxia, allowing metabolic adaptation and the secretion of virulence factors such as staphyloxanthin (a carotenoid which provides protection against ROS) to combat oxidative stress [35,36]. The secretory phenotype also depends on bacterial density, detected by quorum sensing mechanisms. At low densities (e.g., in early infection), *S. aureus* adopts the “adhesive” phenotype, with upregulation of surface adhesion molecules to facilitate attachment and biofilm formation. Later in infection, as determined by local conditions such as hypoxia, staphylococci can switch to an “invasive” phenotype secreting toxins and proteases to promote bacterial dissemination to distant tissues [35,37]. Thus *S. aureus* adjust their expression of virulence factors according to environmental conditions.

## 7. The Impact of Hypoxia on Neutrophil Bactericidal Functions

In vitro studies of human neutrophils suggest that functions underpinning recruitment, such as polarisation and chemotaxis, are fully sustained at low-oxygen levels [38]. Thus a hypoxic environment will not prevent the mobilisation of PMN from circulation in response to infection. This was indeed confirmed in mouse models employing genetic/pharmacological manipulation of the HIF-signalling pathways [39,40] and in animals exposed to acute hypoxia [41]. However, in the whole animal studies, despite equivalent neutrophil recruitment, acute hypoxia dramatically worsened the outcome of the host–pathogen interaction with neutrophil-dependent hypothermia and cardiac compromise, whilst preconditioning animals through longer exposures to hypoxia prior to infection was protective [41]. These complex effects were underpinned by changes in neutrophil metabolism and underscore the need to translate in vitro observations on isolated cells to more clinically relevant systems.

Neutrophil phagocytosis has been reported to be either unchanged [38] or increased [42] by hypoxia. These opposing observations likely reflect methodological differences; Fritzenwanger et al. [42] isolated blood samples from subjects exposed to hypoxia, but isolated the cells in atmospheric oxygen used zymosan as prey. In contrast, McGovern et al. [38] isolated blood from volunteers and then incubated the isolated cells in hypoxic conditions, adding *S. aureus* when the cells had ‘acclimatised’ to hypoxia. However, these studies both provide evidence that hypoxic impairment of neutrophil bactericidal activity does not relate to impaired ingestion of prey.

The function of the NADPH oxidase (defective in CGD) is to generate ROS at the phagosome; together with the discharge of granule proteases, this generates an environment that is hostile to bacterial survival [43]. ROS comprise a range of unstable oxygen free radicals with O_2_•− (superoxide anion), O_2_ (singlet oxygen) and OH• (hydroxyl radical), as well as more stable and freely diffusible radical and non-radical oxidants, such as NO• (nitric oxide) and H_2_O_2_ (generation of the latter radical requiring myeloperoxidase from the neutrophil granules). Molecular oxygen is required for generation of ROS, and is thus an important mediator of neutrophil microbicidal function [44] including the killing of *S. aureus* [38]. Whilst neither genetic nor pharmacologic manipulation of HIF modulated the oxidative burst [39,40], hypoxia sufficient to limit the availability of oxygen as a substrate for the NADPH oxidase can compromise the ability to kill pathogens such as *S. aureus* in an HIF-independent fashion [38]. Thus, it is important to remember that manipulation of HIF in experimental systems does not always recapitulate the effects of hypoxia, particularly in the acute setting.

Granule proteins are key to the effective killing of many pathogens, and hypoxia may modulate degranulation responses in a number of ways. Firstly, neutrophils develop in a hypoxic niche in the bone marrow [45], and HIF-dependent transcription regulates the expression of key bactericidal proteases and peptides including neutrophil elastase, cathepsin G and cathelicidin [39]. Secondly, in vitro exposure to acute hypoxia increased the stimulated release of neutrophil microbicidal proteins and proteases, including myeloperoxidase (MPO), lactoferrin, metalloproteinase-9 (MMP-9) and elastase [30]. This may impact tissue destruction and be relevant to certain aspects of staphylococcal pathology, for example abscess formation (see below). Thirdly, hypoxia may be relevant to the release of neutrophil extracellular traps (NETs), which consist of chromatin decorated with histones and granule proteins and can bind to, restrain and kill both Gram-positive and -negative bacteria [46]. NETosis is a form of neutrophil death, which differs from apoptosis or necrosis and is predominantly ROS-dependent; CGD neutrophils form few NETs with aberrant morphology [47]. Despite this, NET formation in response to viable *S. aureus* (wild-type or nuclease-deficient strains) was retained under hypoxia (1% oxygen), suggesting that even trace levels of ROS suffice to signal NETs release [48] in response to relevant stimuli.

Since hypoxia impairs phagocyte function, exposure to high oxygen tensions has been suggested as a means of promoting infection resolution. Hyperbaric oxygen therapy (HBOT) has been used in a range of experimental and therapeutic settings, including staphylocoocal infection; such systemic hyperoxia will have complex effects on host cells (including phagocytes) and on infecting bacteria. HBOT has been shown to enhance antibiotic efficiency in a rat model of infective endocarditis [49], but not in a mouse model of implant-associated osteomyelitis [50]. The reason for this difference and other conflicting reports in the literature is not clear, but might relate in part to the different model systems and perhaps to the ability of hyperbaric oxygen to increase oxygen tensions locally in vegetations (immediately bathed in oxygenated blood) as opposed to deep within hypoxic bone tissues or other infection sites.

Whilst acute hypoxia modulates neutrophil ROS generation and degranulation responses, more prolonged hypoxia has dramatic effects on neutrophil metabolism and survival. Prolonged stabilisation of HIF1α leads to enhanced glycolytic flux and ATP pools (reviewed in [51]) and diminished neutrophil apoptosis [52]. An important insight was garnered from studying a *S. aureus* murine skin infection in mice placed in different oxygen environments; as noted above, systemic hypoxia converted a minor superficial staphylococcal infection to near-universal with rapid fatality. Enhanced bacterial replication was not the cause of this lethal effect, and preconditioning animals in hypoxia rescued outcomes. The phenotype largely resided in the neutrophil’s response to hypoxia, with HIF1α activation leading to elevated neutrophil glucose requirements, resulting in worse disease outcome in hypoxemic animals such as cardiac failure and hypoglycaemia [41]. Whilst a detailed discussion of such metabolic re-programming in the setting of infection and hypoxia is beyond the scope of this review, the outcomes are pro-inflammatory and usually detrimental to the host.

## 8. The Role of Neutrophils and Hypoxia in Shaping Staphylococcal Infections

Both microbes and immune cells contribute to hypoxia at the site of infection and their response to this environmental pressure helps to shape the progress of infection. As outlined above, hypoxia has a wide and dynamic range of impacts on neutrophil functionality, which may compromise bactericidal function, increase tissue damage and impair inflammation resolution. *S. aureus* possesses a repertoire of environmental sensors and regulatory proteins that interact to detect and respond to low oxygen states, both by activating genes required to handle hypoxic stress and by increasing expression of toxins and proteases (reviewed in [35]). For example, supernatants prepared from *S*. *aureus* induced cytotoxicity in a range of mammalian cells (including neutrophil-like HL-60 cells) that significantly increased if the bacteria were cultured under lower oxygenation [53]. Although the two-component environmental sensor SsrAB was shown to be key to this response, the range of hypoxia-upregulated virulence factors was not further delineated in this study. *S. aureus* increases the transcription of the intercellular adhesin (*ica*) cluster, leading to increased polysaccharide intercellular adhesin (PIA) production and protecting the bacteria against non-oxidative neutrophil killing mechanisms and promoting biofilm formation in anoxic conditions [54]. Lack of available oxygen limits ATP synthesis and results in the inability to replenish NADH/NAD+ pools, restricting growth and promoting the emergence of SCVs [55]. SCVs are enabled with respect to intracellular persistence, antimicrobial resistance, immune evasion and biofilm formation, and have been shown to emerge in the setting of chronic staphylococcal infections [56].

In this section, we describe how hypoxia and the host immune response interact with this organism to cause two typical clinical syndromes of staphylococcal infection, namely abscess formation and infection of prosthetic material.

## 9. Staphylococcal Abscess Formation

Abscesses are the archetypal manifestation of *S. aureus* infections and both host and pathogen factors contribute to their formation (see Figure 2).

Patients with impaired neutrophil function have increased risk of skin infection; patients with Hyper IgE syndrome are particularly prone to staphylococcal skin and lung abscesses [14], although other cellular and cytokine defects may contribute to this susceptibility [57]. Abscess formation is also a frequent consequence of disseminated infection, and in a zebrafish model, such abscesses were shown to arise from small numbers or even single bacteria carried to distant sites whilst residents within host neutrophils [58]. Thus, neutrophils may function as “Trojan” carriers of infection and factors such as hypoxia [37], which favour intracellular persistence, and will encourage such metastatic infection (Figure 2A).

A range of bacterial toxins including Staphylococcal protein A (SpA) and coagulases contribute to early abscess formation [59,60]. Coagulases allow the pathogen to usurp the host coagulation system, forming fibrin deposits which eventually mature to act as a barrier against immune cells, preventing their penetration into the developing abscess [60], where the pathogen replicates without interference. The fibrin capsule matures by cross-linking of components and deposition of collagen. Lysyl oxidase (an amine oxidase required for biosynthetic cross-linking of extracellular matrix components) was found to be upregulated in an HIF-dependent fashion [61]. It is abundantly expressed in human and murine staphylococcal abscesses, especially adjacent to the capsule (although the precise cell type(s) expressing LOX in vivo were not defined). Also, inhibition of LOX reduced collagenisation of the abscess capsule and led to more diffuse infection [61]. These processes are represented in Figure 2B.

Neutrophils are recruited to combat infection, but *S. aureus* secretes a number of leukocidins and haemolysins to lyse neutrophils, and their release may be enhanced in hypoxic settings [61]. Virulence factors such as SarA, which may also contribute to abscess formation, are likewise induced by hypoxia [62]. As infection progresses, the abscess consists of a large mass of pathogens at the central part of the lesion, a layer of necrotic PMNs, followed by other necrotic cells and/or healthy cells and surrounded by a dense fibrin capsule [59,60]. This is a hypoxic environment [25], and in addition to the effects noted on bacteria, hypoxia impairs the capacity to mount a neutrophil respiratory burst to kill ingested *S. aureus* [38]. Also, augmented secretion of proteases by hypoxic neutrophils [30] will contribute to host tissue breakdown and bacterial lysis. Thus, hypoxia acts on both bacteria and host to regulate the establishment and maturation of an abscess cavity (Figure 2C).

## 10. Staphylococcal Biofilms and Infection of Prosthetic Material

**Figure 3:**
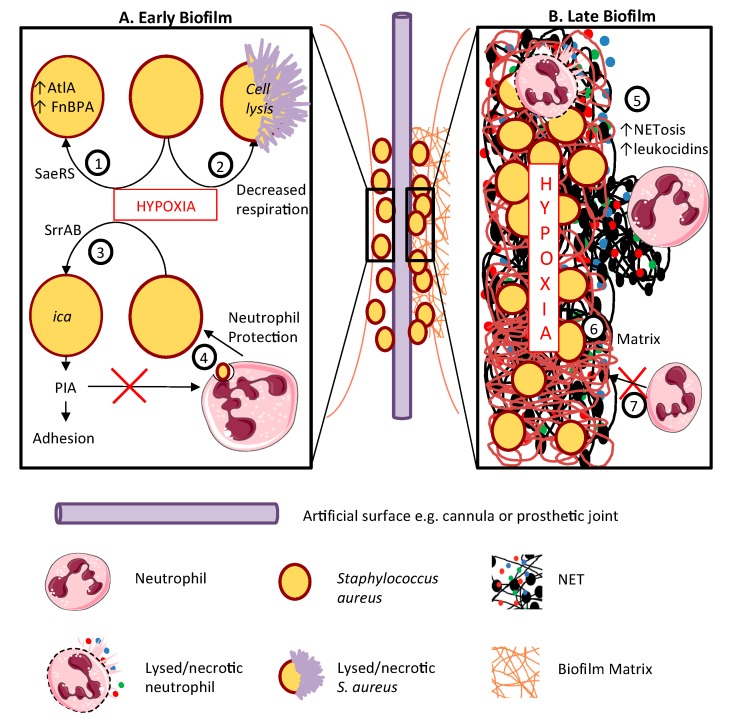
Hypoxia enhances staphylococcal biofilm development. Biofilms are most likely to form on prosthetic surfaces such as intravenous cannulae or replacement joints or heart valves. The host and pathogen processes contributing to biofilm formation are depicted in the left-hand panel **A**. Panel **B** (right) depicts the role of hypoxia in biofilm maintenance and progression. **A.** In early biofilm development, hypoxia activates the SaeRS 2- component system, increasing AtlA and FnBPA production (1); decreases staphylococcal respiration, potentially resulting in cell death and lysis (2); and activates the PIA (polysaccharide intercellular adhesion encoding operon (*ica*) via the SrrAB regulator (3), increasing biofilm adhesion. PIA accumulation also impairs neutrophil non-oxidative killing (4). **B.** Mature biofilms are profoundly hypoxic, promoting NETosis and leukocidin production (5) adding to the polymeric biofilm matrix of polysaccharides, proteins and lipids, entangling multiplying staphylococci (6). This matrix blocks the entry of neutrophils into the biofilm (7). This figure was created using Servier Medical Art templates, which are licensed under a Creative Commons Attribution 3.0 Unported License; https://smart.servier.com.

*S. aureus* has a propensity to cause infections on indwelling prosthetic materials, for example intravenous cannulae, joint replacements and artificial heart valves. Most prosthetic device infections are thought to result from skin flora contamination during implantation or tracking of infection from the subcutaneous portion of the device to deeper sites. The ability of *S. aureus* to form biofilms (surface-associated collections of bacteria embedded within a slimy matrix composed of a polymeric assembly of polysaccharides, proteins, lipids and DNA) is key to its ability to colonise and cause persistent infection of such devices (See Figure 3). Oxygen consumption by microbes and immune cells renders biofilms profoundly hypoxic [63].

Importantly, *S. aureus* increases biofilm formation dramatically in response to hypoxia [64,65]. The mechanism by which hypoxia promotes biofilm formation is imperfectly understood and likely multi-factorial (see Figure 3A). Hypoxic activation of the SaeRS 2-component regulatory system results in increased expression of AtlA (peptidoglycan hydrolase), and fibronectin-binding protein A was shown to be required for biofilm formation, with diminished bacterial respiration leading to programmed cell lysis and promoting biofilm development [64]. The key adhesin responsible for the accumulation phase of Staphylococcal biofilms is the polysaccharide intercellular adhesin (PIA), encoded by the *ica* operon; PIA expression is up-regulated in low oxygen environments under the control of the staphylococcal respiratory response regulator, SrrAB [54].

Established biofilms (Figure 3B) are resistant to antibiotics and to neutrophils [66], skewing these cells towards NETosis, which may encourage biofilm development [10] at least in part by the production of leukocidins, which promote neutrophil lysis and/or NETosis. Interestingly, the outcome of the neutrophil:biofilm interaction depends on its stage of development; in a mouse model, neutrophils conferred early protection against biofilm attachment to implanted devices, but later in the process, IL-1β promoted marked neutrophilic infiltration but increased bacterial load [67]. Thus neutrophils may fail to control or even encourage bacterial growth within the biofilm but prevent local spread or dissemination of infection. In a model of prosthetic joint infection, the interaction of neutrophils with the staphylococcal biofilm led to pro-inflammatory cytokine production, triggering the formation and activation of osteoclasts, bone resorption and prosthesis loosening [68].

## 11. Conclusions

Staphylococcal infections remain a major cause of human morbidity and mortality. Infection and inflammation enhance tissue hypoxia, profoundly influencing the host–pathogen interaction, with both *S. aureus* and neutrophils responding to low oxygen availability. Whilst outcomes will be determined by the precise local conditions, hypoxia may limit bacterial growth and promote bacterial persistence, whilst modulating neutrophil function to engage infection but not to eradicate it. However, such intracellular persistence may, in some settings, allow neutrophils to promote staphylococcal dissemination, and may promote biofilm formation to exclude immune cells from the infective site. Further exploring the molecular mechanisms underlying host–pathogen interactions in the setting of hypoxia may lead to new strategies to treat persistent and recalcitrant infections due to *S. aureus*.

## Figures and Tables

**Table 1:** Examples of *S. aureus* virulence factors relevant to immune evasion.

Group	Virulence Factor	Mechanism
Prevention of phagocyte recognition by opsonisation and hence reduction of phagocytosis	Protein A (SpA)	Cross links Fab domain of IgM and binds Fcγ domain of immunoglobulin G.
	Clumping factor A (ClfA)	Fibrinogen-binding surface protein causing platelet aggregation. Antiphagocytic effect with or without presence of fibrinogen.
	Staphylococcal complement inhibitor (SCIN)	Inhibits C3 complement convertase by preventing the C3b generation.
	Aureolysin	Anti-protease blocks C3 complement activity through cleaving C3 blocking C3a activation of neutrophils. Also cleaves granule-derived antimicrobial peptides.
Induction of phagocyte damage and death	Panton-Valentine leukocidin (PVL) and other leukocidins such as gamma-haemolysin and LukED	Triggers apoptosis and necrosis of cells, initiated by pore formation.
	Phenol-soluble modulins (PSMs)	Cause lysis of blood cells, assists in the structuring and dispersal of biofilms.
	Alpha-haemolysin (alpha toxin)	Forms pores in cells through its interaction with the ADAM10 receptor, resulting in cell lysis.
Prevention of neutrophil chemotaxis and recruitment to sites of staphylococcal infection	Chemotaxsis inhibitory protein (CHIPs)	Blocks chemotaxis towards C5a and formylated peptides by binding to neutrophil C5a receptors formyl peptide receptors, preventing neutrophil recruitment to sites of staphylococcal infection.
	Extracellular adherence protein (Eap)	Blocks complement activation and neutrophil adhesion to activated endothelium inhibiting neutrophil recruitment; suppresses NETosis.
	Staphylococcal superantigen like (SSLs)	A group of structurally similar antigens with functions including binding IgA, IgG, matrix metalloproteinases amd neutrophil adhesion molecules, which act together to inhibit neutrophil recruitment to staphylococcal infection.
Evasion of phagocyte killing	OatA	Catalysing the O-acetylation of peptidoglycan in the Staphylococcal cell wall, rendering it insensitive to lysozyme (which is secreted by phagocytes and constitutively present in secretions such as tears).
	SodA/M	Superoxide dismutases provide resistance to reactive oxygen species (ROS) produced by neutrophils including superoxide.
	Staphyloxanthin	A carotenoid which provides protection against oxidative stress and ROS
	Phenol-soluble modulins (PSMs)	Cause cell lysis, aid in biofilm development and stimulate inflammation.

**Table 2:** Neutrophil Disorders and Infection.

Disease	Defect	PMN Dysfunction	Clinical Outcomes
**Neutropenia**	Decreased PMN numbers, either congenital (e.g., elastase deficiency) or acquired (most commonly drug-induced such as cancer chemotherapy).	Insufficient PMN numbers to respond to invading pathogens, life-threatening Gram-negative and Gram-positive infections.	Life-threatening infections during periods of neutropenia, susceptibility reduced when neutrophil count recovers.
**Chronic granulomatous disease (CGD)**	Mutations in NADPH oxidase components; reduced or absent ROS formation.	Reduced killing of certain pathogens e.g., *Staphylococcus aureus, Aspergillus fumigatus*, Gram- negative bacilli.	Life-threatening infections with *Staphylococcus* and *Apergillus*; aberrant healing (granulomas).
**Hyper IgE Syndrome (formerly Job’s Syndrome)**	Mutations in STAT3 (signal transducer and activator of transcription 3) or DOCK 8 (Dedicator of cytokinesis 8) or TYK2 leading to impaired T cell function and diminished neutrophil chemotaxis	Reduced killing of certain pathogens e.g., *Staphylococcus aureus, Aspergillus fumigatus.*	Staphylococcal and fungal skin infections, pulmonary and joint infections, ‘cold’ abscess formation (reduced cytokine release).
**Myeloperoxidase deficiency**	Decreased or lack of MPO/HOCl system required to generate the full range of ROS.	Increased chronic conditions mediated by adaptive immunity, decreased NET killing of microbes.	Susceptibility to chronic infections caused by *Candida albicans, S. aureus.*
**SGD (Specific Granule Deficiency)**	Absence of specific granules, bilobed neutrophils nuclei. Altered content of other granule populations.	Impaired chemotaxis, aberrant granule organisation, reduced respiratory burst, and deficient bactericidal activity (mainly to *S. aureus*).	Staphylococcal skin infections, aberrant skin lesion healing.
**Chediak Higashi Syndrome**	Mutations in lysosomal trafficking regulator (LYST) leading to failure of lysosomal trafficking in neutrophils and other cells	Giant granules, impaired phagocytosis and phagosomal maturation, oxidative burst and degranulation	Albinism, neurological defects, coagulopathy, recurrent skin (staphylococcal) infections and respiratory infection
